# Prostatic Albuminuria Not an Infrequent Cause of Error in the Diagnosis of the So-called “Orthostatic,” “Postural,” “Physiological” and “Cyclic” Albuminuria

**Published:** 1906-03

**Authors:** Edgar G. Ballenger

**Affiliations:** Lecturer on Genito-Urinary Diseases, Atlanta School of Medicine, Atlanta, Ga.


### ATLANTA
### Journal-Record of Medicine

Successor to Atlanta Medical and Surgical Journal, Established 1855,
and Southern Medical Record, Established 1870.

OWNED BY THE ATLANTA MEDICAL JOURNAL CO.

**Published Monthly.**

Vol. VII. MARCH, 1906. No. 12.

BERNARD WOLFF, M.D., EDITOR, Nos. 319–20 Prudential.

M. B. HUTCHINS, M.D., BUSINESS MANAGER, 1007-1008 Century Bldg.

E. G. BALLENGER, M.D., ASSOCIATE EDITOR AND ASSISTANT MANAGER.

J. N. LECONTE, M.D., FOREIGN CORRESPONDENT.

### *ORIGINAL COMMUNICATIONS.*

### PROSTATIC ALBUMINURIA NOT AN INFREQUENT CAUSE OF ERROR IN THE DIAGNOSIS OF THE SO-CALLED "ORTHOSTATIC", "POSTURAL", "PHYSIOLOGICAL", AND "CYCLIC" ALBUMINURIA.*

By EDGAR G. BALLENGER, M.D.,

Lecturer on Genito-Urinary Diseases, Atlanta School of Medicine,
Atlanta, Ga.

It is a well-known fact that albumin in the urine does not always indicate a kidney lesion, and I think that in the following paper the facts show that the albuminous prostatic secretion, from glands that are hyperemic or chronically inflamed, is not an infrequent source of error in many of the cases of comparatively harmless albuminurias.

From many examinations of urine, passed after massage of the prostate, even when only slightly diseased, in which a proteid material, giving the tests for albumin, was constantly found, it

* Read before the Fulton County Medical Society.

occurred to me that this might be a more or less frequent cause of confusion as to the significance of albuminuria, and also a reliable sign as to the integrity of the prostate.

There can be no doubt that this fluid, when expressed by muscular exertions or from excessive secretions, passes back into the bladder much more frequently than it appears at the meatus in the form of a prostatorrhea. In routine massage of the prostate not more than one patient in eight or ten will have the secretion discharge from the urethra, while the urine passed after massage always contains albumin and prostatic debris. If the inflammation is well marked it will contain large flaky masses or shreds. The character of the secretion and the amount of the albumin vary of course with the severity of the condition.

All or various combinations of the following substances may be present: Serum albumin, globulin(?), nucleo-albumin (occasionally in considerable quantities), mucin, epithelial cells, leucocytes, microorganisms, phosphates (very abundant), amyloid bodies (rather rare), and occasionally hyaline casts similar to but slightly larger than those of renal origin. Spermatozoa may or may not be present, and unless they are there is nothing in the urine containing the prostatic secretion that will enable one to determine the source of the albumin, either by a chemical test or by a microscopical examination.

In doubtful cases, therefore, the prostate and the seminal vesicles should be massaged thoroughly, the bladder irrigated and emptied, and the urine examined from the second glass of a specimen passed in about thirty minutes. This method will at least remove one source of contamination with extrarenal albumin, and may explain many cases of the harmless albuminuria that are so frequently encountered and have made it an unreliable sign of kidney disease.

Much pains is taken to avoid uterine and vaginal discharges in women, but the prostate is entirely disregarded unless there be an acute inflammation or a profuse prostatorrhea, which the patient thinks is a loss of semen.

Urine containing this secretion may be clear, slightly cloudy, like very liquid glue, or milky in appearance. This white cloudiness may clear up on the addition of a few drops of nitric acid. The heat test for albumin is often not satisfactory, owing to the alkalinity of the prostatic fluid, which may prevent the formation of a precipitate. If the urine is not acid enough to overcome this, dilute acetic acid must be added, but drop by drop or it will become too acid and form a soluble acid albumin. For this reason much care is required in making the heat test. Nitric or picric and citric acid will throw down a precipitate after boiling that gradually settles to the bottom and gives an idea as to the amount of albumin present.

The cold nitric acid test (Heller's) shows a distinct white ring at the zone of contact with the urine.

Robert's solution, as modified by Boston, containing one part of nitric acid to ten parts of a saturated solution of magnesium sulphate, does not have the dark ring of oxidation at the junction of the fluids that occurs with undiluted nitric acid, and showed the presence of albumin whenever it was demonstrated by any other method.

The picric acid test was nearly always positive, as was potassium ferrocyanide when used as a layer test.

The first urine passed by patients with a very slight watery urethral discharge sometimes will show the presence of a small amount of albumin, consequently the specimen for examination should always be from the second portion of urine.

The secretion from the prostate of one patient being passed with only about 5c.c. of urine was nearly clear until an equal quantity of water was added; this produced a precipitate of large white masses, probably of nucleo-albumin which is insoluble in water but is held in solution by the salts of the urine. It may be detected by the addition of acetic acid, which produces a white cloud. It responds to nearly all the tests for serum-albumin and is very similar to mucin.

The mucleo-albumin probably is derived from the disintegration of the cells lining the prostate ducts and follicles, while the serum-albumin more than likely exudes from the inflamed or desquamated surfaces.

Now, having shown the prostate a possible source of albumin, I

desire to call attention to the striking similarity in the causes and symptoms of postural or orthostatic albuminuria and the flow of the secretion from diseased prostates. Both come on after exertions in the upright position, and are usually worse in the latter part of the day. Neither have renal casts, except perhaps hyaline, which are not characteristic of any disease. The extensive studies of Dr. A. W. Stirling have shown that postural albuminuria is far more frequently found in the male than in the female. Neither of these diseases cause much impairment of health. No vascular changes are found in either. Phosphates are likely to be abundant in both. Oxalate of calcium may be present in both; two patients showed an excessive amount in the urine after massage of the prostate.

It is characteristic of the so-called "cyclic", "postural", etc., albuminuria that the amount of albumin is small in quantity and intermittent. In regard to this last feature, will relate some facts as to the periodic increase in the secretion from the prostate that has been strikingly apparent in five patients with chronic prostatitis. This observation occurred long before I thought there might be any relation between it and albuminuria. So marked is this in some patients that they fear they are to have a relapse and occasionally it is so regular that the time for an increase can be correctly predicted.

Most of the patients were given a prostatic massage every two or three days, and less frequently as they improved.

Upon the dates given below there was a distinct increase of debris massaged from the prostate. The amount of this increase varied from one to five times the amount obtained at the treatments during the intervals and lasted from two to five days. As the patients improved the amount of the increase diminished and the intervals became longer.

CASE 1. Age 38, married, had gonorrhea fifteen years, double epididymis, acute prostatitis, and chronic urethritis. I assumed charge during acute prostatitis and as it subsided and became chronic there was a distinct increase in the secretion on the following dates: June 20; June 30 (slight increase); July 8 (slight); August 14 (much, lasting 5 days); September 7 (much); October 30 (moderate); November 6 (considerable, lasting 5 days); November 21 (much); December 1 (much); December 7 (little); December 29 (many large dense masses); January 18 (considerable).

CASE 2. Greek, age 35, married, tailor. Complaint, urethral discharge. Prostate enlarged twice normal size; stricture of large caliber 3 inches from the meatus. Increase in prostatic fluid: March 27; April 11; April 25; May 1; May 18 (much); June 13 (little); June 19 (very slight); July 7 (very little).

CASE 3. Age 33, married, gonorrhea twice, mild prostatitis during last attack. Marked increase in secretion massaged from the prostate: September 13; September 25 and 27; December 8; January 1 (only cloudiness); January 22 (only cloudiness).

CASE 4. Clerk, age 25, single. Had gonorrhea four years ago, lasted six weeks, sexual weakness, varicocele, chronic prostatitis and vesiculitis. Increase in discharge massaged from prostate and vesicles: June 16; June 30 (4 days); July 10; July 20; August 14 (5 days); August 25; September 5 (much); September 25. Fresh attack of gonorrhea interrupted the history here.

CASE 5. Age 34, single, gonorrhea ten years ago, chronic prostatitis, urethritis, and stricture. Increase in amount from prostate: August 5 (much); August 18 (less); September 9; October 7; November 6; November 20; December 28.

The tests for albumin were many times positive after the masses had entirely disappeared from the debris massaged from the prostate and its presence seems to be a very reliable indication of the condition of the prostatic follicles. When the patients are nearly well *it* also may occur only at periods.

In trying to explain the reason for a periodic increase in the secretion of a diseased prostate it occurred to me that there might be some relationship between it and menstruation in women, as the prostate is the analogue of the uterus. No inference of course could be drawn from so few cases, but I hope others may look out for this periodic increase so as to determine by longer series of patients if it is constantly present. The peculiar relapses sometimes seen in gonorrheal urethritis that occur without any apparent cause may perhaps be due to this periodic wave of congestion or whatever it is.

I have a patient on hand now who was told five years ago in New York that he had serious kidney disease and would probably not live more than a year. Upon assuming charge of this patient there was a large quantity of albumin in his urine and he also had a marked prostatitis. At no time were there any symptoms of renal disease nor were there any casts present. I can not say what was found by the doctor who made the diagnosis of nephritis, but it is not improbable that it might have been due to prostatic albumin, for since treating him the albumin has entirely cleared up, except after massage of his prostate, then it is constantly found. I believe there are more patients than we realize with conditions similar to the one just described and that we should take more care in eliminating the prostatic secretions in reaching our conclusions.

The points I wish to emphasize are:

1. Diseased prostates like diseased kidneys secrete albumin. The secretion from the normal gland is not albuminous, or it is present in such a small quantity that, unless the seminal vesicles are massaged, the urine passed will not give the tests for albumin.

2. This is apparently a constant symptom of chronic prostatitis and may be depended upon in the diagnosis.

3. *Prostatic albuminuria* seems to be an appropriate name for this condition.

4. In making insurance examinations as well as in the diagnosis of obscure forms of albuminuria this possibility should be eliminated with the other sources of contamination before reaching a positive conclusion.

5. The periodic increase in the prostatic discharge, along with the striking similarity between the symptoms of "intermittent," "postural," "orthostatic" and "cyclic" albuminuria, and prostatorrhea makes the possibility of mistakes in the diagnosis extremely likely when this fluid flows back into the bladder and does not appear at the meatus.

6. This regular increase every 10 to 30 days, and the analogy between the uterus and the prostate, suggest a relationship between the causes of this condition and menstruation.

DISCUSSION OF DR. BALLENGER'S PAPER.

*Dr. W. E. Wilmerding:* This is a very important paper, but I can not see where it will apply to, or in any way affect insurance, as an applicant will never allow you to massage his prostate. I have seen nothing whatever in the latest works on the subject.

*Dr. W. B. Armstrong:* This is an important paper, as I believe it is original work. There is one point I would like to make as to the differential diagnosis between postural albuminuria and prostatic albuminuria. In the postural condition we have renal symptoms, headache, swelling of hands, etc.

*Dr. M. B. Hutchins:* I have had an opportunity of following Dr. Ballenger in his work and have watched its development with great pleasure. I think there is a great deal in this subject and consider that the doctor has made out a good case.

*Dr. Claude Smith:* This is an interesting subject and we are indebted to Dr. Ballenger. We must be careful to remember not to confound this prostatic condition with postural albuminnria.

*Dr. Bernard Wolff:* Did I understand from the doctor that albumin was found only in cases of diseased prostate? Has he ever found albumin in the urine after massaging the normal prostate? I believe Dr. Stirling's investigations were chiefly among boys in whom it would not be likely for the prostate to be diseased.

In regard to the analogy between the prostatic gland and the uterus, it is possible that the phenomenon of sexual periodicity in the male may be explained on the assumption of secretory variations in the prostate.

*Dr. W. B. Goldsmith:* I wish to add a word of admiration to Dr. Ballenger for his excellent paper, and on the line of Dr. Wolff there is a question if there is not a certain periodic exacerbation or "rutting" period with man and at such times albumin may appear in the urine. Dr. Ballenger deserves credit for his paper.

*Dr. E. C. Thrash:* I think that the analogy between the prostate gland and the uterus is rather far-fetched. The erotic period is psychic in my opinion.

*Dr. E. G. Ballenger* (in closing): In answer to Dr. Wilmerding, I will say that I had a case under my care with prostatic albuminuria who had been refused insurance and after treating his prostate he has since obtained insurance. In answer to Dr. Wolff will say that the normal gland does not give off albumin—the seminal vesicles do, but in exceedingly small quantity.

I agree with Dr. Goldsmith that the prostate may affect certain periodicity in regard to sexual passion or excitement. I also believe that the prostate is analogous to uterus.

